# Bisphenol-C is the strongest bifunctional ERα-agonist and ERβ-antagonist due to magnified halogen bonding

**DOI:** 10.1371/journal.pone.0246583

**Published:** 2021-02-09

**Authors:** Xiaohui Liu, Keitaro Suyama, Takeru Nose, Miki Shimohigashi, Yasuyuki Shimohigashi

**Affiliations:** 1 Laboratory of Structure-Function Biochemistry, Department of Chemistry, Faculty of Science, Kyushu University, Fukuoka, Japan; 2 Department of Applied Microbial Technology, Faculty of Biotechnology and Life Sciences, Sojo University, Kumamoto, Japan; 3 Faculty of Arts and Science, Kyushu University, Fukuoka, Japan; 4 Division of Biology, Department of Earth System of Science, Faculty of Science, Fukuoka University, Fukuoka, Japan; 5 Risk Science Research Institute, Fukuoka, Japan; University of Parma, ITALY

## Abstract

We reported that bisphenol AF (BPAF) works as an agonist for estrogen receptor (ER) ERα but as an antagonist for ERβ. Similar results were observed for bisphenol E analogs (BPE-X) such as BPE-F, BPE-Cl, and BPE-Br, each consisting of a series of a tri-halogenated methyl group CX_3_ in the central alkyl moiety. It was demonstrated that the electrostatic halogen bond based on the dispersion force of halogen atoms is a major driving force in the activities of bifunctional ERα-agonist and ERβ-antagonist. Since the chlorine atoms present in bisphenol C (BPC) exist in a π-π conjugated system due to the presence of an adjacent C = C double bond, we intended to prove that BPC is also a bifunctional ERα-agonist and ERβ-antagonist exhibiting greatly enhanced agonist/antagonist activities. BPC was evaluated for its ability to activate ERα and ERβ in the luciferase reporter gene assay using HeLa cells. With high receptor-binding ability to both ERs, BPC was found to be fully active for ERα but inactive for ERβ. BPC’s definite antagonist activity in ERβ was revealed by its inhibitory activity against 17β-estradiol. Thus, BPC is a bifunctional ERα-agonist and ERβ-antagonist. These agonist/antagonist activities were discovered to be extremely high among series of halogen-containing bisphenol compounds. This comparative structure-activity study revealed that the ascending order of ERα-agonist and ERβ-antagonist activities was BPE-F ≪ BPE-Cl ≲ BPAF < BPE-Br ≪ BPC. The highly intensified receptor interaction of BPC is attributable to the presence of an n-π-π-n conjugation system mediated through the >C = CCl_2_ double bond.

## Introduction

**Fig 1:**
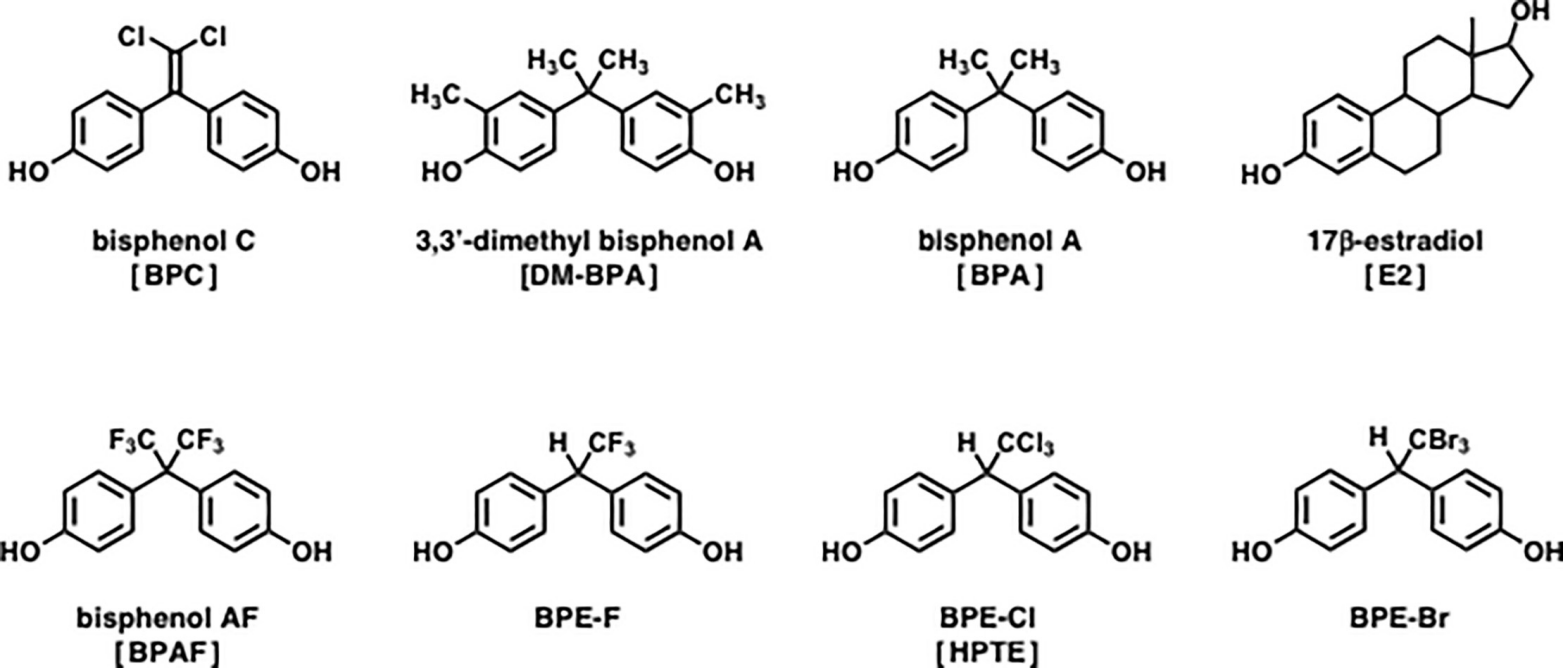
Chemical structures of the bisphenol compounds examined for estrogen receptor ERα and ERβ. 3,3’-Dimethyl bisphenol A (DM-BPA) is often and confusingly labeled “bisphenol C (BPC)”, and was not tested in this study. 17β-Estradiol (E2) was tested as a standard natural agonist compound.

Bisphenol A (BPA) (Fig 1), an endocrine-disrupting chemical, has been reported to be unfavorable to human health, especially in fetuses, infants, and children [1–5]. The adverse effects intrinsic to BPA appear to occur by the toxicity in signalling through nuclear receptors (NRs). To avoid the hazards of BPA, various kinds of BPA-free products made of so-called next-generation (NextGen) bisphenol compounds have become increasingly important [6, 7]. However, almost none of these NextGen bisphenols have been evaluated for their signalling toxicity. Based on the critical importance of receptor-binding evaluation for human NRs, we have recently evaluated BPA and its 10 NextGen analogs for their abilities to bind to 21 NRs, the greatest members of NRs for which tritium-labeled specific ligands were available [8]. It is now important to assess such NextGen bisphenols for their abilities to activate or inhibit the NRs.

The target NRs of BPA include estrogen receptors (ERs) [4, 8–12], which are members of the transcriptionally active nuclear receptor family [13–15]. Two ERs exist, ERα and ERβ, and they are genetically and physiologically independent from each other. Although there are distinct differences in the protein structures of ERα (595 amino acids [aa]) and ERβ (530 aa), the 3D structures of their DNA-binding domain (DBD) and ligand-binding domain (LBD) are very similar [16, 17]. Estrogen-related receptor γ (ERRγ) has been reported as the most likely major target NR of BPA [9, 10, 12]. Including other estrogen-related receptors ERRα and ERRβ, all of these NRs associated with the term ‘estrogen’ appear often to exist simultaneously in some of the same cells [18–22]. As with the relationships between or among NRs, the reactivities of exogenic non-intrinsic compounds to those NRs are very important, especially in order to understand their cellular responsiveness [1, 23].

17β-Estradiol (E2) (Fig 1), a natural steroid hormone, binds to both ERα and ERβ [24, 25]. BPA acts as an agonist for these two ERs but works much more weakly than E2 [3, 11, 12, 26]. We previously reported that bisphenol AF (BPAF) (Fig 1), a NextGen bisphenol, binds to ERs 30–50 times more strongly than BPA, while it acts as an agonist for ERα but as an antagonist for ERβ [27]. BPAF has two CF_3_ groups in the central bisphenol-connecting moiety. Similar curious results of ERα-agonist/ERβ-antagonist activities were observed for the compound denoted as HPTE, which consists of a CCl_3_ group in the central alkyl moiety of its bisphenol bone structure (Fig 1) [28, 29]. The reason why BPAF and HPTE exhibit dual ERα-agonist and ERβ-antagonist activities has recently been uncovered by a structure-activity study of their activities in conjunction with those of chemically synthesized CF_3_- and CBr_3_-containing bisphenol E analogs (BPE-F and BPE-Br, respectively: Fig 1) [30]. HPTE is also a bisphenol E analog and was thus designated also as BPE-Cl (Fig 1).

BPAF, HPTE or BPE-Cl, BPE-F, and BPE-Br, all of which are bisphenols comprised of a tri-halogenated methyl group (CX_3_), showed the ERα-agonist/ERβ-antagonist dual biological activities [30]. The ascending order of such agonist/antagonist dual activities was BPE-F < BPE-Cl (HPTE) ≤ BPAF < BPE-Br, revealing that the electrostatic halogen bonding effect is a major driving force in bifunctional ERα agonist and ERβ antagonist activities [30]. Halogen bonding, a kind of electrostatic interaction, is dependent on the dispersion force of halogen atoms [31]. It is very intriguing that similar bonding effects characterize completely different receptor responses of the same bisphenol compound; i.e., agonist activity for ERα but antagonist activity for ERβ. It should be noted that, as a precondition of antagonist activity, these bisphenols containing one or more CX_3_ groups were almost completely inactive for ERβ.

Bisphenols consisting of one or more halogen atoms at the molecular terminus in the central moiety are thus a probable candidate exhibiting ERα-agonist/ERβ-antagonist bifunctional activities. Bisphenol C (BPC) (Fig 1) is unique in that it has two chlorine (Cl) atoms at the geminal positions on the C = C double bond carbon in the central moiety of the bisphenol structure. BPC has been used in the production of fire-resistant polymers [32, 33], although it has been assumed to be a strong candidate for an endocrine disruptor, as is BPA. Although the BPC molecule has no CX_3_ group, its characteristic structural feature is the presence of a conjugation system between the C = C double bond and the aromatic benzene rings of phenol groups. Since such an expanded π-electron network would result in the reinforcement of electron delocalization, these structural characteristics would greatly enhance the dispersion force of Cl atoms. The objective of this study is to examine whether BPC is also a compound with ERα-agonist/ERβ-antagonist dual or bifunctional activities and, if so, to validate the idea that its C = C double bond adjacent to the Cl atoms would increase the dispersion force to strengthen the receptor interactions.

Here is another important note about BPC. Note that a considerable number of reports have labeled 2,2-bis(4-hydroxy-3-methylphenyl)propane (CAS no. 79-97-0) as “bisphenol C (BPC)” (Fig 1), causing a lot of confusion especially in bibliographic searches and in the selection of appropriate distinguishing assay data. This compound is designated as 3,3’-dimethyl bisphenol A (DM-BPA) in PubChem, an open chemistry database of the National Center for Biotechnology Information (NCBI) of the National Institutes of Health (NIH) [34]. In the DM-BPA molecule, the two phenol-phenyl groups (namely, the benzene rings) are methylated simultaneously at the position of 3, which is the *ortho* position of the 4-hydroxy group, and this is why 2,2-bis(4-hydroxy-3-methylphenyl)propane is designated as 3,3’-dimethyl bisphenol A in PubChem. DM-BPA has no Cl atom, but its central part maintains exactly the same 2,2-disubstituted-propane structure as in BPA (Fig 1). In the present study, according to the PubChem nomenclature, BPC is used only for our target compound 1,1-bis(4-hydroxyphenyl)-2,2-dichloroethylene [35] as shown in Fig 1, and not for DM-BPA. We strongly recommend adopting the PubChem nomenclature to discriminate BPC from DM-BPA.

## Materials and methods

### Chemicals

E2 or 17β-estradiol (CAS no. 50-28-2; 98.9% purity) was purchased from Research Biochemicals International (Natick, MA, USA). BPA, 2,2-bis(4-hydroxyphenyl)propane (CAS no. 80-05-7; >99% purity) and BPAF, 2,2-bis(4-hydroxyphenyl)hexafluoropropane (CAS no. 1478-61-1; >98% purity), were purchased from Tokyo Chemical Industry (TCI) (Tokyo, Japan). BPC (CAS no. 14868-03-2; 98% purity) was purchased from Sigma-Aldrich (St. Louis, MO, USA). BPE-Cl, or HPTE, 2,2-bis(4-hydroxyphenyl)-1,1,1-trichloroethane (CAS no. 2971-36-0; >98% purity), was also obtained from Sigma-Aldrich. BPE-F and BPE-Br were obtained from stock chemically prepared in our laboratory previously [30].

### Receptor-binding assays for estrogen receptors ERα and ERβ

#### Preparation of GST-fused estrogen receptor LBD protein

The glutathione *S*-transferase (GST)-fused receptor ligand-binding domains (LBDs) of ERα (amino acid residues 301 to 595) and ERβ (242 to 530), namely, GST-ERα-LBD and GST-ERβ-LBD, were prepared essentially by using the expression construct of ERα-LBD/pGEX-6p-1 and ERβ-LBD/pGEX-6p-1 as described previously [8]. Briefly, these receptor proteins were expressed in *E*. *coli* BL21α and purified on an affinity column (10×100 mm) of Glutathione Sepharose 4B (GE Healthcare BioSciences, Piscataway, NJ, USA) followed by gel filtration on a column of Sephadex G-10 (15×100 mm; GE Healthcare BioSciences). The purity was confirmed by SDS-PAGE using 12.5% polyacrylamide gel and by staining with Coomassie brilliant blue (CBB). The protein concentrations were determined by the Bradford method [36].

#### Radio-ligand binding assays for saturation binding

We first performed saturation binding assays to ensure the quality of the purified receptor proteins. These assays were conducted essentially as described previously [8, 27, 37] at 25°C for 1 h. The assay conditions established for this study were as follows. GST-ERα-LBD or GST-ERβ-LBD (60 ng) was incubated with 1–10 nM tritiated ligand [^3^H]17β-estradiol ([^3^H]E2) (5.74 TBq/mmol; Amersham Biosciences, Buckinghamshire, UK) in a final volume of 100 μl of binding buffer [10 mM Tris-HCl (pH 7.4), 1 mM ethylenediaminetetraacetic acid (EDTA), 1 mM ethylene glycol-bis(β-aminoethylether)-*N*,*N*,*N*’,*N*’-tetraacetic acid (EGTA), 1 mM NaVO_3_, 10% glycerol, containing 2 mg/mL γ-globulin].

Nonspecific binding was evaluated using [^3^H]E2 together with nonlabeled E2 (final concentration: 10 μM) to quantify the specific binding by subtracting the nonspecific binding from the total binding. For the bound/free (B/F) separation, free [^3^H]E2 was removed by filtration after incubation with 0.4% dextran-coated charcoal (DCC; Sigma-Aldrich) in phosphate-buffered saline (PBS; pH 7.4) for 10 min at 4°C. The DCC-adsorbed free [^3^H]E2 was eliminated by the direct vacuum filtration method using a 96-well filtration plate (MultiScreen^HTS^ HV, 0.45-μm pore size; Millipore, Billerica, MA, USA). Radioactivity was determined on a liquid scintillation counter (LS6500; Beckman Coulter, Fullerton, CA, USA).

The data on receptor populations showing the appropriate dissociation constant (*K*_d_) and the receptor density (*B*_max_) were used for the following competitive receptor-binding assay. The specific binding data of [^3^H]E2 were first assessed by means of a Scatchard plot analysis [38] to ensure that the binding of [^3^H]E2 to the GST-ER-LBDs is merely one-site ligand binding. The data were then applied to a one-site binding hyperbola nonlinear regression analysis using the software package Prism 8 (GraphPad Software, La Jolla, CA, USA) to measure changes in the values of *B*_max_ and *K*_d_. The saturation receptor-binding assay was performed at least three times for every preparation of GST-ER-LBDs to verify GST-ERα-LBD or GST-ERβ-LBD to warrant its use in the next competitive binding assays.

#### Radio-ligand binding assays for competitive binding

Compounds were dissolved in *N*,*N*-dimethylsulfoxide (DMSO) and diluted by 2 mg/mL γ-globulin with a half-logarithmic 3.16-fold dilution method, keeping the DMSO concentration below 0.3%. γ-Globulin, but absolutely not bovine serum albumin (BSA), was used as a blocker of nonspecific adsorption to the plasticware. Bisphenols were examined for their ability to inhibit the binding of [^3^H]E2 (final concentration: 1 nM) to GST-ERα-LBD (60 ng) or GST-ERβ-LBD (60 ng). The assay solutions were incubated at 25°C for 1 h, and B/F separation was carried out by the DCC method as described above. Radioactivity was determined on a liquid scintillation counter (TopCount NXT; PerkinElmer Life Sciences Japan, Tokyo).

To estimate the binding affinity, the IC_50_ values (half maximal inhibitory concentrations) were estimated from the dose-response curves generated by GraphPad Prism 8. Each assay was performed in duplicate and repeated at least three times.

### Luciferase reporter gene assay for transcription activation activity

HeLa cells (RCB0007; RIKEN BRC, Koyadai, Tsukuba, Japan) were maintained in Eagle’s Minimum Essential Medium (Nissui, Tokyo) in the presence of 10% (v/v) fetal bovine serum at 37°C in a 5% CO_2_ atmosphere. For the luciferase assays, HeLa cells were seeded at 5×10^5^ cells/6-cm dish for 24 h and then transfected with 3 μg of reporter gene (pGL3/3 × estrogen response element [ERE] (a kind donation from the Chemicals Evaluation and Research Institute, Tokyo, Japan)) and 1 μg of ERα- (NM_000125.4; nucleic acid residues 232 to 2019) or ERβ-full length (NM_001437.2; 469 to 2061) expression plasmid (pcDNA3.1/ERs) by Lipofectamine LTX reagent (Invitrogen Japan, Tokyo) according to the manufacturer’s protocol.

Approximately 24 h after transfection, the cells were harvested and plated into 96-well plates at 5×10^4^ cells per well. The cells were then treated with varying doses of chemicals diluted with 1% BSA in PBS (v/v). Compounds were initially dissolved in neat DMSO and then diluted with 1% BSA (<0.3% DMSO final concentration) by the half-logarithmic 3.16-fold dilution method. Here, instead of γ-globulin, BSA was used as a blocker of nonspecific adsorption to the plasticware.

Twenty-four hours later, the luciferase activity was measured with the appropriate reagent by the use of a Luciferase Assay System according to the manufacturer’s instructions (E1500; Promega, Madison, WI, USA). Light emissions were measured using a multilabel counter (Wallace 1420 ARVOsx; PerkinElmer). Cells treated with 1% BSA/PBS were used as a vehicle control. Each assay was performed in triplicate and repeated at least three times.

### Luciferase reporter gene assay for transcription inhibitory activity for ERβ

In the *in vitro* luciferase reporter gene assay to measure qualitatively the antagonistic activity of bisphenols for ERβ, a serial concentration of bisphenols (10^−13^ to 10^−5^ M in the final solution) was assayed in the HeLa cells in the presence of 10 nM E2, which elicits a full activation of ERβ. Each assay was performed exactly as described above for transcription activation activity. In order to measure quantitatively the antagonistic activity of bisphenols for ERβ, four different concentrations (0.01, 0.1, 1.0, and 10 μM) of the respective bisphenol were examined for a serial concentration of 17β-estradiol (10^−13^ to 10^−5^ M in the final solution).

### Calculation methods

#### Gaussian calculation

The molecular orbital calculation was carried out using the Gaussian-16 series program, which provides advanced capabilities for electronic structure modeling. The total atomic charge value was obtained for each atom of the central moiety of connecting bisphenols, and then the respective dipole moments were calculated.

#### Molecular structure optimization

Molecular structure optimizations of the bisphenol compounds were carried out in order to draw the van der Waals surface by using Discovery Studio 2019 software. The optimizations were performed by the “clean geometry” command, and the molecular volume of each central moiety was estimated.

#### Statistical analysis

Data are presented as means ± SD for the indicated number of separate experiments. The significance of differences was determined by the two-sided Student’s *t*-test.

## Results

### Receptor binding activities of BPC for ERα and ERβ

**Fig 2:**
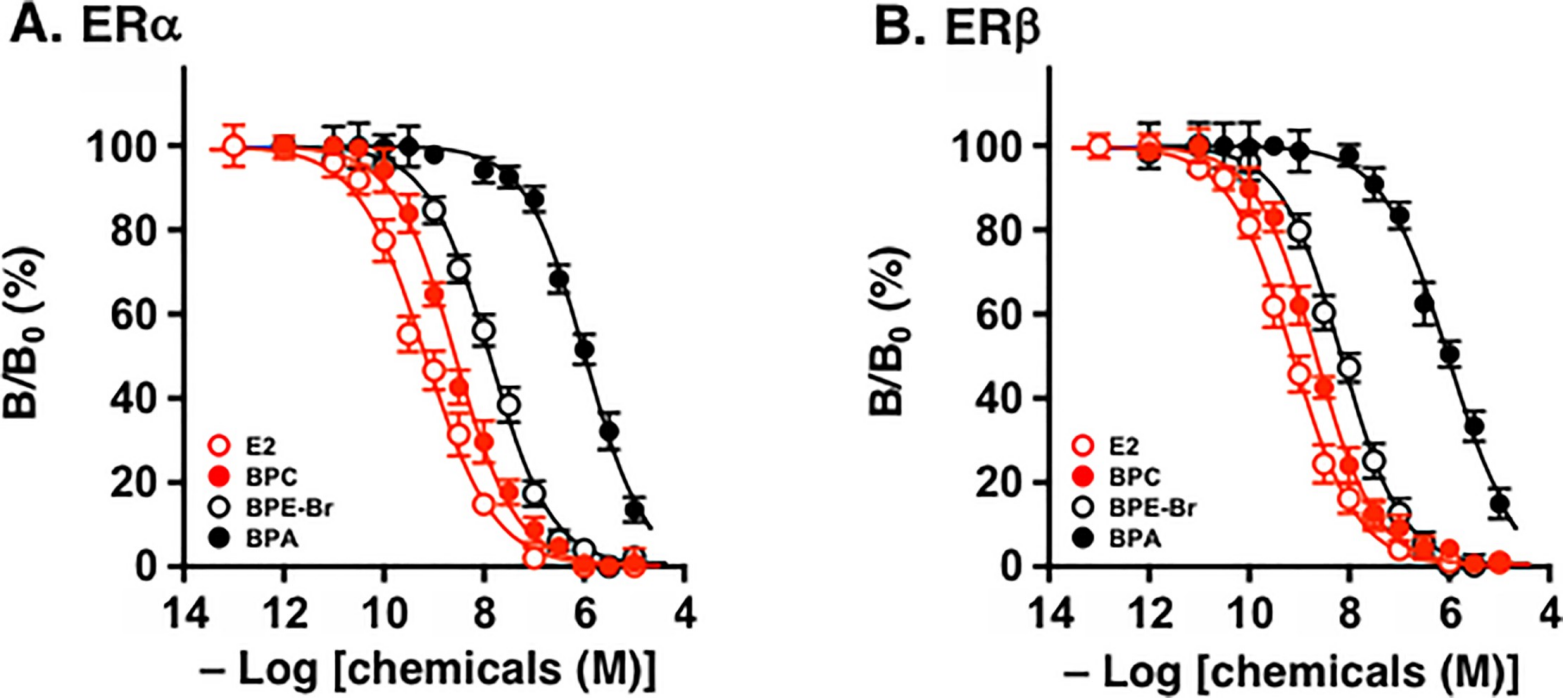
Competitive radio-ligand receptor-binding assays. Dose-response receptor binding curves are shown for the assays for (**A**) ERα and (**B**) ERβ. 17β-estradiol (E2) was used as an internal standard compound in the assays for both ERα and ERβ, in which tritiated [^3^H]E2 was used as a radiolabeled receptor tracer. The Y axis is expressed by the normalized binding data from 100% (no competitor chemical) to 0% (nonspecific binding at maximal concentrations of competitor). Log[chemicals (M)] is the logarithm of the concentration of competitor chemicals plotted on the X-axis.

BPC is unique in that it has two Cl atoms at the geminal positions on the C = C double bond carbon (Fig 1). BPC was generated chemically by the dehydrochlorination of HPTE or BPE-Cl (i.e., 2,2-bis(4-hydroxyphenyl)-1,1,1-trichloroethane) [32, 39], which forms an *sp*^2^ coplanar structure from an *sp*^3^ tetrahedral structure. We observed that this prominent structural difference between BPC and BPE-Cl (HPTE) induced a major activity difference in the binding assays for estrogen receptors ERα and ERβ. Compared to BPE-Cl (HPTE), BPC became extremely highly active (Fig 2, Table 1). BPC was approximately 17 times more active than BPE-Cl (HPTE) for ERα and approximately 8 times more active than BPE-Cl (HPTE) for ERβ (Table 1).

**Table 1:** Receptor-binding affinities and selectivities of the bisphenol compounds.

Bisphenols	Receptor binding affinity IC_50_ (nM)		Receptor binding selectivity^1)^
	ERα	ERβ	ERβ over ERα
E2^2)^	0.70 ± 0.038	0.73 ± 0.065	0.96
BPA^2)^	1085 ± 35.2	1014 ± 66.6	1.07
BPC	2.65 ± 0.05	1.94 ± 0.11	1.37
BPAF	43.6 ± 2.72	14.4 ± 0.71	3.03
BPE-F	197 ± 4.35	93.8 ± 4.16	2.10
BPE-Cl^3)^	45.1 ± 2.18	15.9 ± 0.76	2.84
BPE-Br	14.2 ± 1.04	7.78 ± 0.12	1.83

1) The selectivity ratio was calculated by dividing the IC_50_ value obtained for ERα by that for ERβ.

2) 17β-Estradiol (E2) was used as a natural standard ligand for both ERα and ERβ. BPA was used as the standard bisphenol.

3) BPE-Cl is exactly the same compound as HPTE (see Fig 1).

Since the binding potency of BPC itself was very high, with nanomolar-level IC_50_ values (half-maximal inhibitory concentrations) of 2.65 nM for ERα and 1.94 nM for ERβ (Table 1), the presence of the >C = CCl_2_ moiety in the bisphenol backbone of BPC’s structure appears to be very preferable for binding to ERs. Although the ER natural ligand 17β-estradiol (E2) was several times more potent than BPC, BPC’s very strong receptor-binding affinity was enough to pervert BPC just like a natural ligand of ERs. The potency of E2 was extremely high with subnanomolar-level IC_50_ values for both ERα and ERβ (Table 1).

BPC was much more potent than BPA; i.e., it was approximately 410-fold more potent for ERα (Fig 2A) and 520-fold more potent for ERβ (Fig 2B). These results closely replicated our previous findings [8, 30]. BPAF was only slightly more potent than BPE-Cl (HPTE), and it was thus revealed that BPC was approximately 16 times more potent than BPAF for ERα and approximately 7 times more potent than BPAF for ERβ (Fig 2, Table 1).

Our recent results revealed that, among the halogen-containing BPE analogs, the ascending order of receptor-binding affinity for ERα was BPE-F < BPE-Cl (HPTE) < BPE-Br. This result was replicated very well in the present study, as shown in Table 1. The IC_50_ values were 197 nM, 45.1nM, and 14.2 nM, respectively, clearly proving that BPC (2.65 nM) is much stronger than any of these bisphenols containing a CX_3_-group. BPC was approximately five times more active than even the strongest BPE-Br for ERα. Similar results were observed in the radio-ligand receptor-binding assay for ERβ, for which BPC (1.94 nM) was approximately four times more active than the most potent BPE-Br (7.78 nM: Table 1).

### Receptor binding selectivity of BPC for ERα and ERβ

The receptor-binding selectivity ratio for ERβ over ERα was estimated by dividing the IC_50_ value obtained for ERα by that for ERβ (Table 1). E2 and BPA were found to be almost completely nonselective for ERβ versus ERα, showing selectivity ratios of 0.96 and 1.07, respectively. All the CX_3_-containing bisphenols—BPAF, BPE-F, BPE-Cl (HPTE), and BPE-Br—revealed a favorable selective ratio for ERβ versus ERα.

BPAF consists of the two CF_3_ groups in the central bisphenol-connecting moiety (Fig 1), and it was the first bisphenol compound whose bifunctional ERα-agonist/ERβ-antagonist activities were noted [27]. The ERβ-versus-ERα selectivity ratio of BPAF was 3.03 (Table 1), implying that BPAF prefers ERβ to ERα almost three times more. The structural lack of one of the two CF_3_ groups, creating another bisphenol compound, BPE-F (Fig 1), resulted in decreased receptor-binding affinity for both ERα and ERβ. However, since the extent of the decrement was larger for ERβ (6.5-fold) than for ERα (4.5-fold), the ERβ-versus-ERα selectivity ratio dropped slightly (3.03→2.10).

When the chemical structures of the CX_3_-containing bisphenols were compared, BPE-Cl (HPTE) was found to have a CCl_3_ group instead of the CF_3_ group in BPE-F, while BPE-Br was found to have a CBr_3_ group. These replacements increased greatly the receptor-binding affinities for both ERα and ERβ. In the case of BPE-Cl (HPTE), the extents of the increases were greater for ERβ (5.9-fold) than for ERα (4.4-fold), resulting in a slight increase in the ERβ-versus-ERα selectivity ratio (2.10→2.84). In the case of BPE-Br, however, the extent of the increase was greater for ERα (13.9-fold) than for ERβ (12.1-fold). This made BPE-Br only slightly more selective than ERβ (selectivity ratio = 1.83).

BPC exhibited high receptor-binding affinities for both ERα and ERβ with nanomolar levels of IC_50_ values (Table 1). As for this highly potent bisphenol compound BPC, the receptor-binding selectivity ratio for ERβ over ERα showed that BPC is only slightly selective for ERβ, with a selectivity ratio of 1.37 (Table 1). Since BPE-Cl (HPTE) is a starting material of BPC as described below, we compared their receptor-binding affinities. BPC was approximately 17 and 8 times more potent than BPE-Cl (HPTE) for ERα and ERβ, respectively (Fig 2, Table 1). Thus, it was revealed that the α,β-dehydrochlorination of BPE-Cl to create BPC increased the receptor-binding affinity for ERα more effectively.

### Transcription activation activities for ERα and ERβ

**Fig 3:**
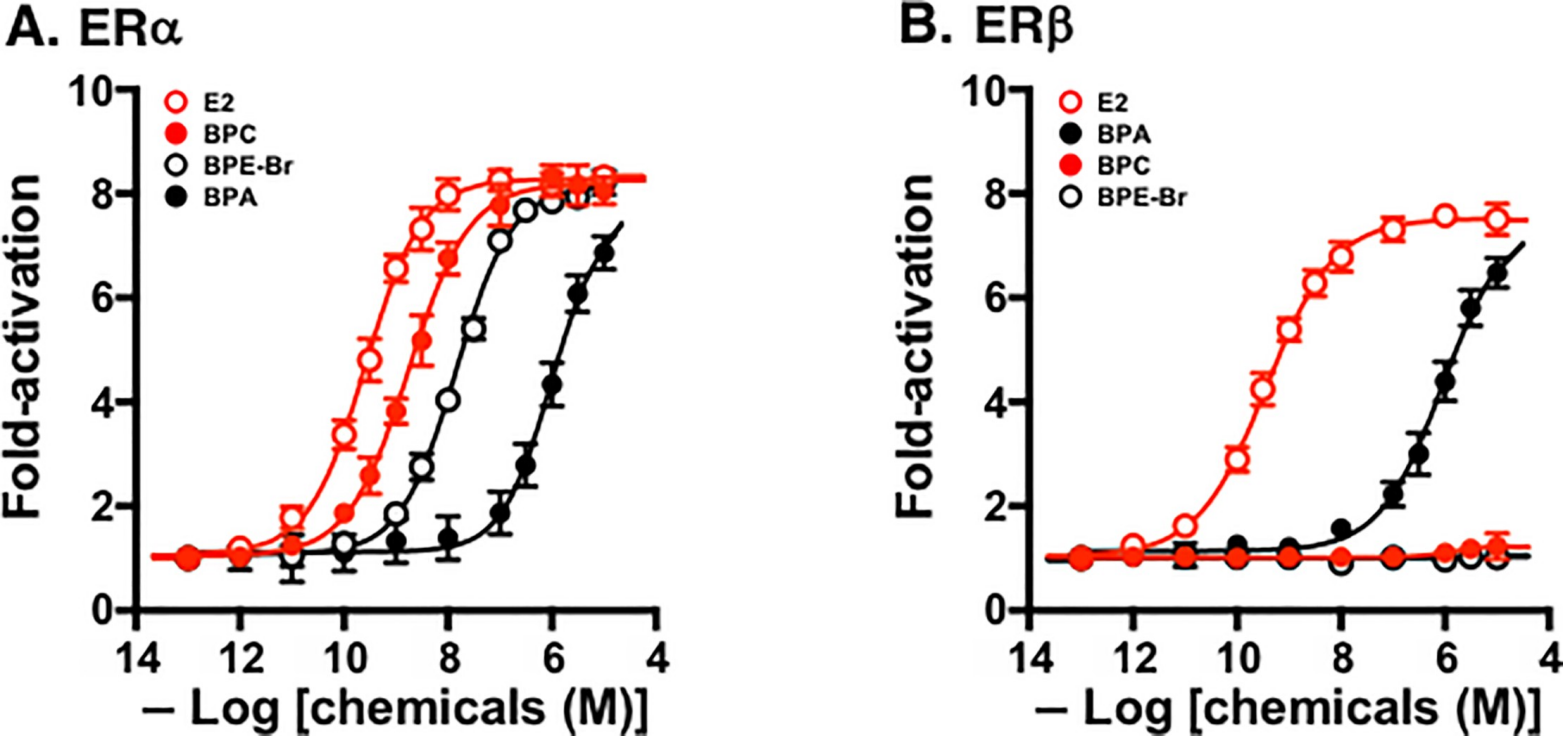
Luciferase reporter gene transcription activation assays. Dose-response curves are shown for the assays for (**A**) ERα and (**B**) ERβ. E2 was used as an internal standard compound in the assays for ERα and ERβ. Log[chemicals (M)] is the logarithm of the concentration of test chemicals plotted on the X-axis.

BPC, an excellent ER binder, was then evaluated for its ability to activate ERα and ERβ in the luciferase reporter gene assay, which was performed using HeLa cells transiently expressing the full-length ERα or ERβ. For ERα, BPC functioned as a full agonist (Fig 3A). Its EC_50_ value (the effective concentration sufficient to induce a half-maximal 50% effect) was at the nanomolar level of 2.20 nM (Table 2). Thus, BPC was recognized as a compound that works as a highly potent transcriptional activator for ERα. In contrast, BPC never activated ERβ at any concentration (Fig 3B). BPC was almost completely inactive for ERβ.

**Table 2:** Transcriptional activity of bisphenols for estrogen receptors ERα and ERβ.

Bisphenols	Transcriptional activities			
	Activation activity EC_50_ (nM)		Inhibitory activity for ERβ	
	ERα	ERβ	IC_50_ (nM)	pA_2_^1)^
E2^2)^	0.181 ± 0.010	0.316 ± 0.027	—	—
BPA	996 ± 93.3	910 ± 66.9	no inhibition	no inhibition
BPAF	28.7 ± 1.50	inactive	61.5 ± 3.56	8.04 ± 0.07
BPC	2.20 ± 0.18	inactive	6.76 ± 0.21	8.96 ± 0.15
BPE-F	106 ± 3.04	almost inactive^3)^	335 ± 14.9	7.46 ± 0.24
BPE-Cl^4)^	39.3 ± 2.08	inactive	84.3 ± 3.86	8.03 ± 0.37
BPE-Br	13.1 ± 1.47	inactive	35.2 ± 4.88	8.36 ± 0.033

1) pA_2_ is a measure of the antagonist’s affinity for a receptor. pA_2_ = –Log *K*_B_, where *K*_B_ is the dissociation equilibrium constant of the antagonist for the receptor.

2) 17β-estradiol (E2) was utilized as a natural standard ligand to measure the transcription activation or inhibitory activity for both ERα and ERβ.

3) BPE-F was extremely weakly active (up to 20%) for ERβ at the 0.1–10 μM concentration.

4) BPE-Cl is exactly the same compound as HPTE (see Fig 1).

Similar activity features, namely, full agonist activity for ERα but inactivity for ERβ, have been reported recently for a series of CX_3_-containing BPE analogs, including BPE-Cl (HPTE) and BPE-Br [30], and for BPAF [27]. These features were reconfirmed also in the same luciferase reporter gene assays in this study, and BPE-Br was found to be the most potent ERα-agonist. When compared with these CX_3_-containing bisphenol compounds, BPC was found to function as a full activator or agonist even more strongly than the most potent BPE-Br for ERα (Fig 3A, Table 2). Regarding activity for ERβ, CX_3_-containing bisphenols were almost completely inactive (Table 2). The only exception was BPE-F, as reported [30]. BPE-F, an extremely weak partial agonist for ERβ, activated ERβ only slightly (up to 20%) at very high concentrations (0.1–10 μM).

The natural steroid hormone E2 fully and very strongly activated both ERα and ERβ (Fig 3). Since BPC, unlike E2, was inactive for ERβ (Fig 3B), it was highly suspected that the structural elements of the BPC molecule differ considerably from those of E2. The inactivity of BPC means that BPC consists of certain structural elements that put ERβ in an inactivation conformation. Since the ‘parent bisphenol compound’ BPA was fully active for ERβ (Fig 3B) as well as E2, such structural element(s) must be in the central halogen-containing moiety, which connects the two phenol groups. Thus, the 2,2-dichloroethylene moiety (>C = CCl_2_) must be intrinsic in order to interact antagonistically with ERβ.

### Inhibitory action of BPC for E2 agonist activity

**Fig 4:**
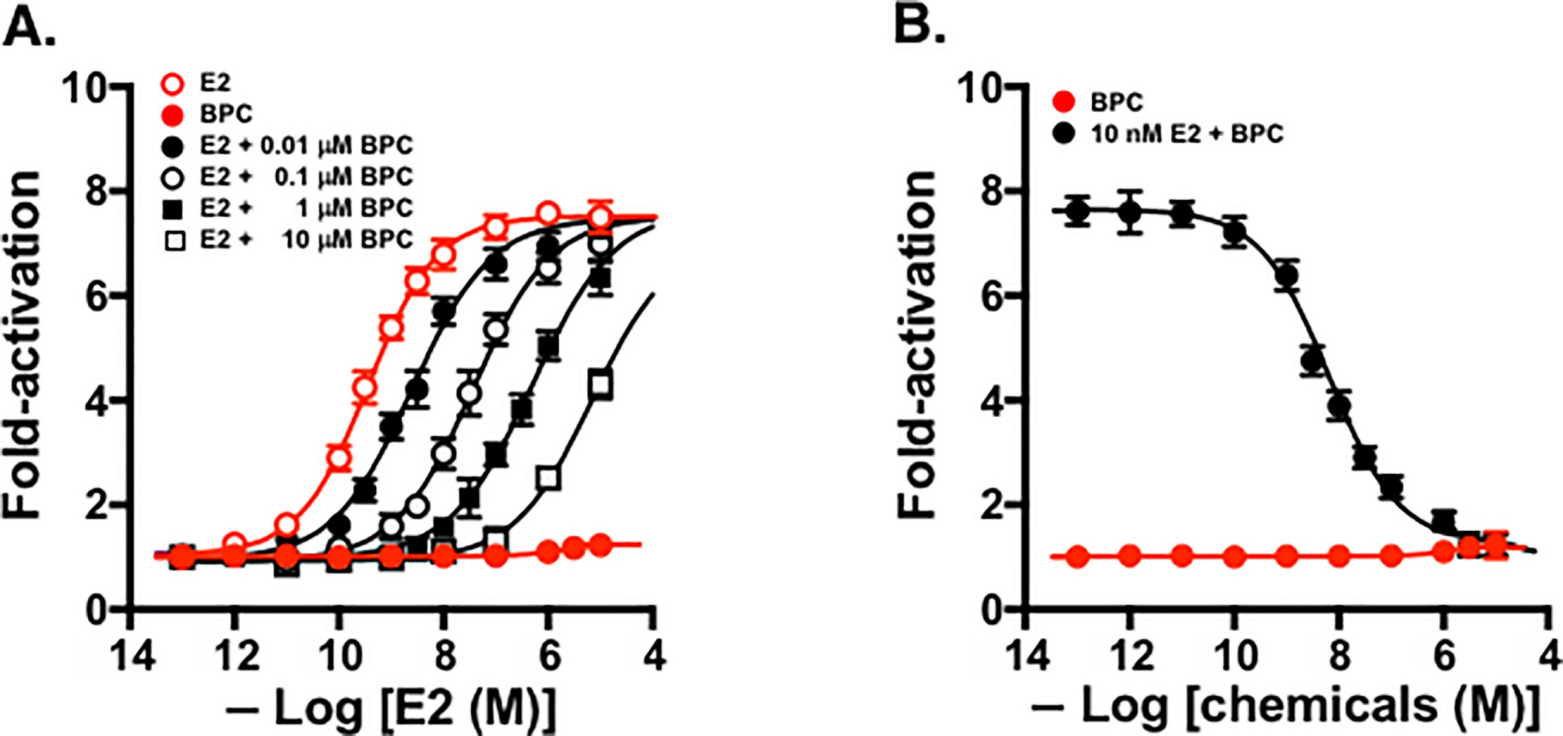
Luciferase reporter gene transcription inhibition assays for ERβ. The dose-response curves are shown for (**A**) the residual activity of 10 nM E2 for ERβ in the presence of serial concentrations of BPC, and (**B**) E2 from the assays for ERβ in the presence of serial concentrations of BPC.

Our observation that, despite its very high receptor-binding potency, BPC did not activate ERβ led us to speculate that BPC must work as a robust antagonist. We thus examined BPC’s ability to inhibit the ERβ natural agonist E2. The examination was carried out by two established methods. First, BPC’s antagonist activity was verified qualitatively in the assay in which serial concentrations of BPC (10^−13^ to 10^−5^ M) were added to a solution of E2 maintained at a constant concentration. E2 exhibited approximately 95% transcriptional activity at its 10 nM concentration for ERβ. When 10 nM E2 was treated with BPC, the activity of E2 decreased in a dose-dependent manner in response to the BPC concentration (Fig 4A). From this dose-response curve, the apparent IC_50_ value was estimated to be 6.76 nM (Table 2). These results demonstrated clearly that BPC could antagonize the activity of E2 on ERβ.

Regarding bisphenols containing tri-halogenated methyl CX_3_ group(s), such as BPAF, BPE-Cl (HPTE), BPE-Br, and even BPE-F, their antagonist activities were also proved for 10 nM E2 in ERβ as described above. The assessed IC_50_ values were 61.5 nM (BPAF), 84.3 nM (BPE-Cl), and 35.2 nM (BPE-Br) (Table 2). The apparent IC_50_ value of BPE-F was 335 nM (Table 2), implying that BPE-F exhibited the weakest activity among the bisphenols examined. More important was that BPC inhibited E2/ERβ most effectively in this analysis.

### Quantitative antagonist activity evaluated by Schild plot analysis

Next, the transcriptional activity of E2 was tested quantitatively in the presence of BPC at different concentrations: 0.01, 0.1, 1.0, and 10 μM. As shown in Fig 4B, the dose-response curves of E2 shifted to the right (i.e., to the higher-concentration regions) as the concentrations of BPC increased, and thus the activity of E2 (EC_50_ = 0.316 nM) gradually weakened (2.54 nM, 36.9 nM, 510 nM, and 6,880 nM). These results demonstrated that BPC effectively inhibits the interaction between E2 and ERβ.

Since this inhibition by BPC was clearly dose-dependent, we examined the results shown in Fig 4B by conducting a Schild plot analysis [40, 41]. The calculated value of pA_2_, a measure of an antagonist’s affinity for a receptor, was 8.96 (Table 2) from the dissociation equilibrium constant (*K*_B_ = 1.10 × 10^−9^ M). As reported previously [30], similar ERβ-antagonist results were obtained also for BPAF, BPE-Cl (HPTE), and BPE-Br, revealing the pA_2_ values of 8.04, 8.03, and 8.36, respectively (Table 2). All of these bisphenols were much weaker than BPC.

When BPE-F, a weak partial agonist, was tested for its antagonist activity against ERβ, the basal transcription activity of E2 was elevated slightly at the concentration range of 10^−13^ to 10^−10^ M, as observed previously [30]. As a result, BPE-F exhibited a mixed effect of weak agonist and antagonist actions for E2/ERβ, showing apparent estimates of *K*_B_ and pA_2_ values (Table 2).

One of the characteristic features of BPC’s biological activity was its full antagonistic activity against ERβ. BPC was practically completely inactive for ERβ, and it functioned as a full antagonist of E2. On the other hand, Delfosse et al. reported that BPC was a partial agonist of ERβ in their assay, revealing an activation capability with approximately 35% of the transactivation activity of 10 nM E2 [42]. BPC was thus observed to show only a weak antagonist effect. Although the exact reasons for these discrepancies are not known, a notable dissimilarity between the two studies was our present use of HeLa reporter cells. Delfosse et al. used the HELN cell line, which was generated by stable transfection with an estrogen-responsive reporter gene and full-length human ERβ gene into the HeLa cells [42]. In contrast, we used HeLa cells transiently expressing an estrogen-responsive reporter gene and the ERβ expression plasmid. This could have resulted in, for example, different recruitments of coactivator proteins, which could have markedly influenced their interactions with ER proteins. Also, we should be cautious especially with regard to the possible existence of endogenous nuclear receptors other than ERβ, which might interact with BPC to bring about a reinforced receptor response beyond our expectations in the cells.

## Discussion

### Orders of relative activity of halogen-containing bisphenol compounds

The ascending activity orders for CX_3_-containing BPA analogs were reported to be BPE-F < BPE-Cl (HPTE) ≤ BPAF < BPE-Br [30]. Those include the orders for receptor-binding affinity (IC_50_) for ERα, agonistic activation activity (EC_50_) for ERα, receptor-binding affinity (IC_50_) for ERβ, and antagonistic activity (pA_2_ and IC_50_) for ERβ. The structural differences between these CX_3_-containing BPA analogs are −CF_3_ (BPE-F), −CCl_3_ (BPE-Cl or HPTE), 2 × −CF_3_ (BPAF), and −CBr_3_ (BPE-Br), holding exactly the same additional bisphenol backbone structure as the two phenol groups (Fig 1). It should be noted that the size of halogen atom X and the numbers of electrons increase down a group; i.e., F < Cl < Br. Thus, the activity ranking proved to correlate well with interacting strength.

Other significant issues were: (i) all these CX_3_-containing BPA analogs were conformationally very rigid due to the presence of bulky CX_3_-group(s) on the *sp*^3^ bisphenol central carbon; (ii) BPAF was much more potent than BPE-F; and (iii) BPAF was slightly more active than BPE-Cl. Eventually, the activity orders were established to be BPE-F < BPE-Cl ≤ BPAF < BPE-Br [30].

In the present study, the receptor potency of BPC was newly uncovered together with its activity ranking among all the CX_3_-containing BPA analogs. These bisphenol compounds were evaluated simultaneously on the same assays (Tables 1 and 2), and here the results were calculated for their specific activities relative to the activity of BPE-Br (Table 3). BPC and CX_3_-containing BPA analogs, namely, BPAF, BPE-F, BPE-Cl (HPTE), and BPE-Br, exhibited good correlations between receptor-binding affinity and biological transcription activation or inhibition activity for ERα and ERβ. The orders of specific activities in all cases were BPE-F <BPE-Cl < BPAF < BPE-Br (100) < BPC. It should be noted again that BPC is the most active compound that works as a highly potent transcriptional activator for ERα and also as a highly potent transcriptional inhibitor for ERβ. When we took the average of the activity magnification values (Table 3), the ascending order of the activity levels was judged clearly and characteristically to be BPE-F ≪ BPE-Cl ≲ BPAF < BPE-Br ≪ BPC.

**Table 3:** Specific activities of halogen-containing bisphenols for ERα and ERβ.

Bisphenols	ERα agonist		ERβ antagonist			Average receptor activity
	Receptor binding affinity	Activation activity	Receptor binding affinity	Inhibitory activity	Antagonist dissociation constant^1)^	
BPE-F	7.2	12	8.3	11	13	10
BPE-Cl^2)^	31	33	49	42	47	40
BPE-Br^3)^	100	100	100	100	100	100
BPAF	33	46	54	57	48	48
BPC	536	595	401	521	397	490

1) Original *K*_B_ values estimated for the specific activity were 34.7 nM (BPE-F), 9.33 nM (BPE-Cl), 4.37 nM (BPE-Br), 9.12 nM (BPAF), and 1.10 nM (BPC).

2) BPE-Cl is exactly the same compound as HPTE (see Fig 1).

3) As for the specific activity, the activity of BPE-Br was referred to as the standard (= 100).

**Fig 5:**
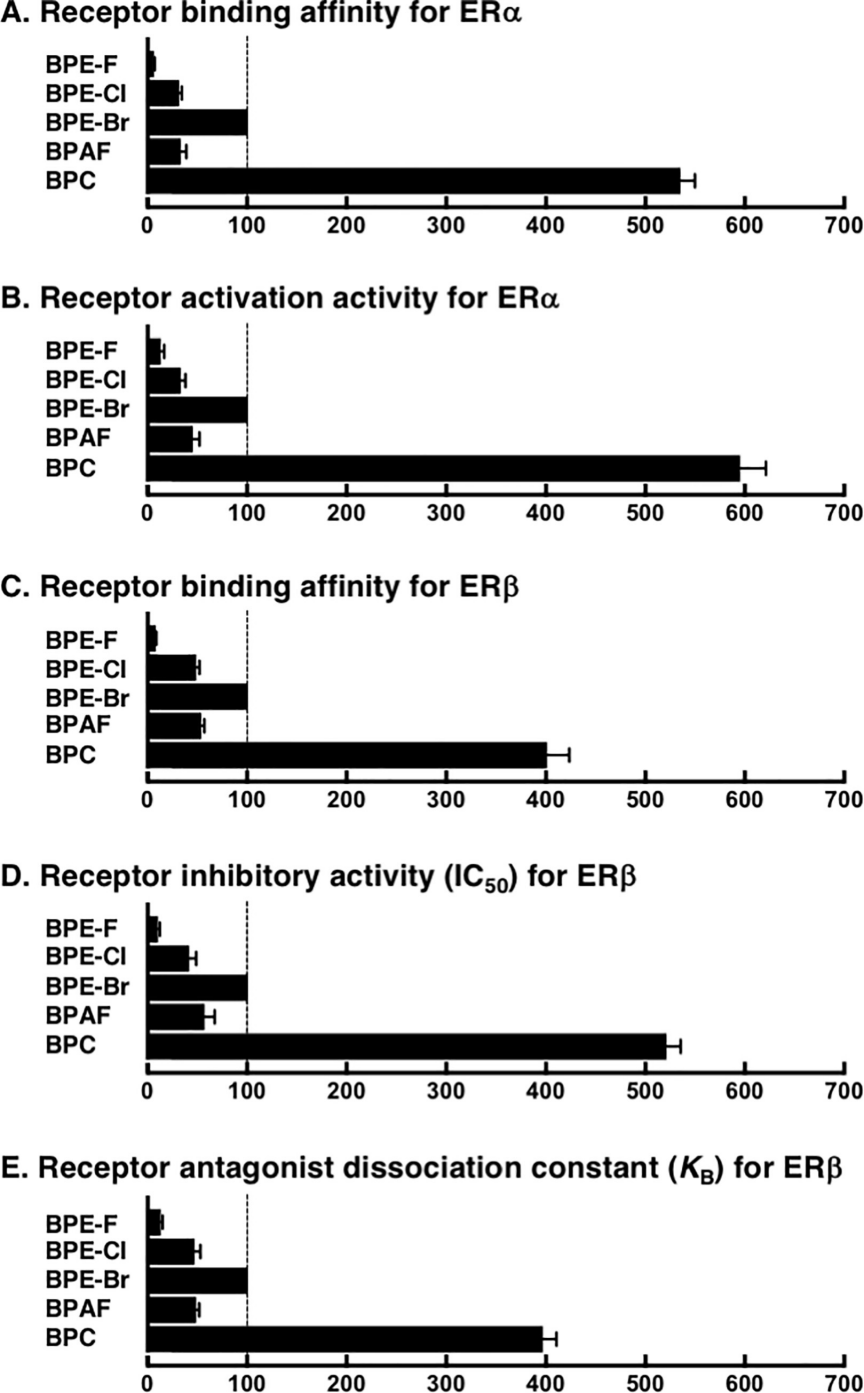
The bar chart diagrams of specific activities among halogen-containing bisphenols. The activities of the bisphenols in the receptor-binding assays and luciferase reporter gene assays for ERα and ERβ were estimated by referring to the activity of BPE-Br as the standard (= 100). BPE-Cl is exactly the same compound as HPTE (see Fig 1).

To illustrate the specific activities for a straight comparison, a bar chart diagram representing each receptor response is displayed in Fig 5. The bar chart diagrams easily identify the ascending order of specific activities of bisphenol compounds in all the assay cases. Eventually, the ascending order of the activity levels is assessed and concluded to be BPE-F ≪ BPE-Cl ≲ BPAF < BPE-Br ≪ BPC. BPC is by far the strongest bisphenol compound in any of the assays carried out.

### BPE-Cl → BPC dehydrochlorination effect

**Fig 6:**
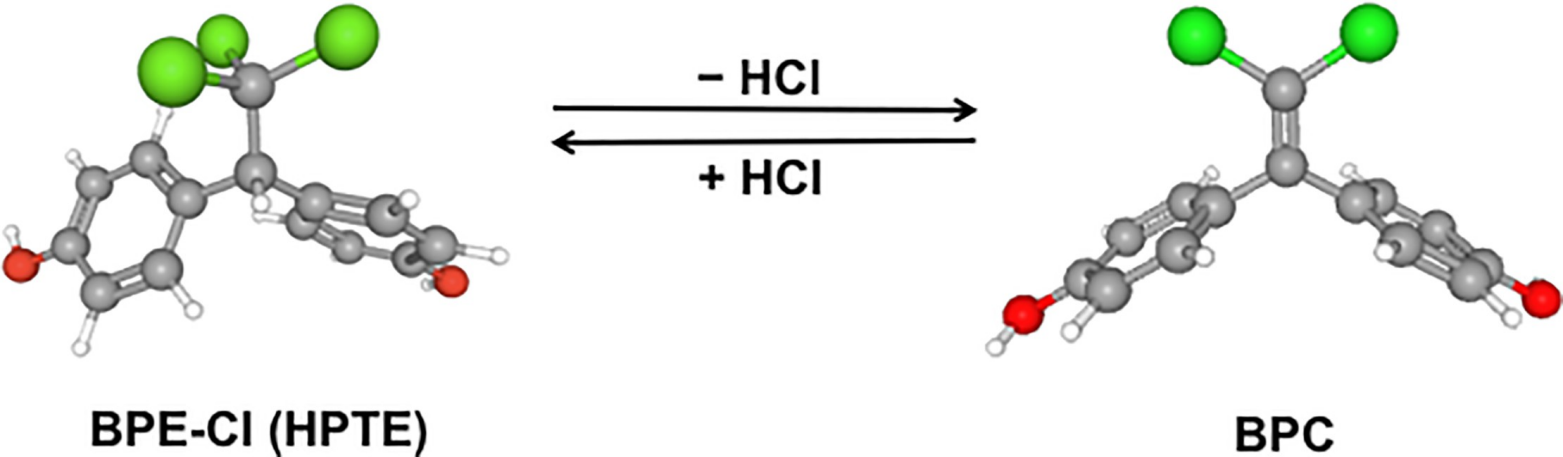
Dehydrochlorination (–HCl) and hydrochlorination (+ HCl) between BPC and BPE-Cl (HPTE). This chemical reaction formula is shown only for form’s sake. Synthesis of BPC by the dehydrochlorination of BPE-Cl (HPTE) was described by Cleveland et al. [39]. Ball and stick models are shown. The 3D-conformers of BPE-Cl [43] and BPC for this reaction formula were obtained from the chemical information resource PubChem [35].

Bisphenol compounds BPC and BPE-Cl (HPTE) are in the relationship of being hydrochlorinated or dehydrochlorinated. The effect of dehydrochlorination, or HCl exclusion, between BPE-Cl and BPC (Fig 6) on the enhancement of receptor activities of BPC was found to be great. The scale factors of BPE-Cl≪ BPC were 17.3-fold in the receptor-binding affinity for ERα, 18.0-fold in the receptor activation activity for ERα, 8.2-fold in the receptor-binding affinity for ERβ, 12.4-fold in the inhibitory activity (IC_50_) for ERβ, and 8.4-fold in the dissociation constant (*K*_B_) for ERβ. These activity enhancements were enough to lead us to suspect that the BPC molecule has some special structural and electronic effectiveness.

### Halogen bonding as a crucial driving force for receptor activity

What type of driving force determined the activities of the halogen-containing bisphenols? To answer to this question, we examined the structure-activity relationships of assayed bisphenol compounds to compare their physico-chemical molecular characteristics [30]. It should be noted that structural differences among these bisphenol compounds occur only in the central bisphenol-connecting moieties (Fig 1). In the present study, newly including the compound BPC, those structural differences were re-evaluated in detail for the results obtained in the receptor-binding assays and the luciferase reporter gene assays for ERα and ERβ. First, taking the ordinary van der Waals forces into consideration, we compared the molecular volumes of the central bisphenol-connecting moieties. After the molecular structure was optimized, it was found that the order of *estimated molecular volumes* is BPE-F < BPC < BPE-Cl < BPAF < BPE-Br. The most potent bisphenol, BPC (96.77 Å^3^), was found to have a rather compact volume compared to BPE-Cl (117.12 Å^3^), BPAF (124.44 Å^3^), and BPE-Br (130.70 Å^3^), all of which were far weaker than BPC (Table 3, Fig 5). These results strongly indicated that the BPC molecule is under the influence of some special effects to increase the van der Waals force.

What is a special effect on the BPC molecule? When the atom charges of central bisphenol-connecting moieties were calculated by the computer-assisted molecular orbital calculation method GAUSSIAN, the estimated distributed electron densities were (0.186, 0.186) for C*Cl*_*2*_ of BPC. The dipole moment was 0.455 D, the direction of which was outward from the molecule. Including these BPC data, all the molecular data [30] were re-evaluated and compared for all the halogen-containing bisphenol compounds: BPE-F, BPE-Cl, BPAF, BPE-Br, and BPC. However, the results showed no relevance to explain an ordered receptor interaction or activity at all. Thus, neither electron densities nor dipole moments were found to explain the ordered receptor interaction or activity.

**Fig 7:**
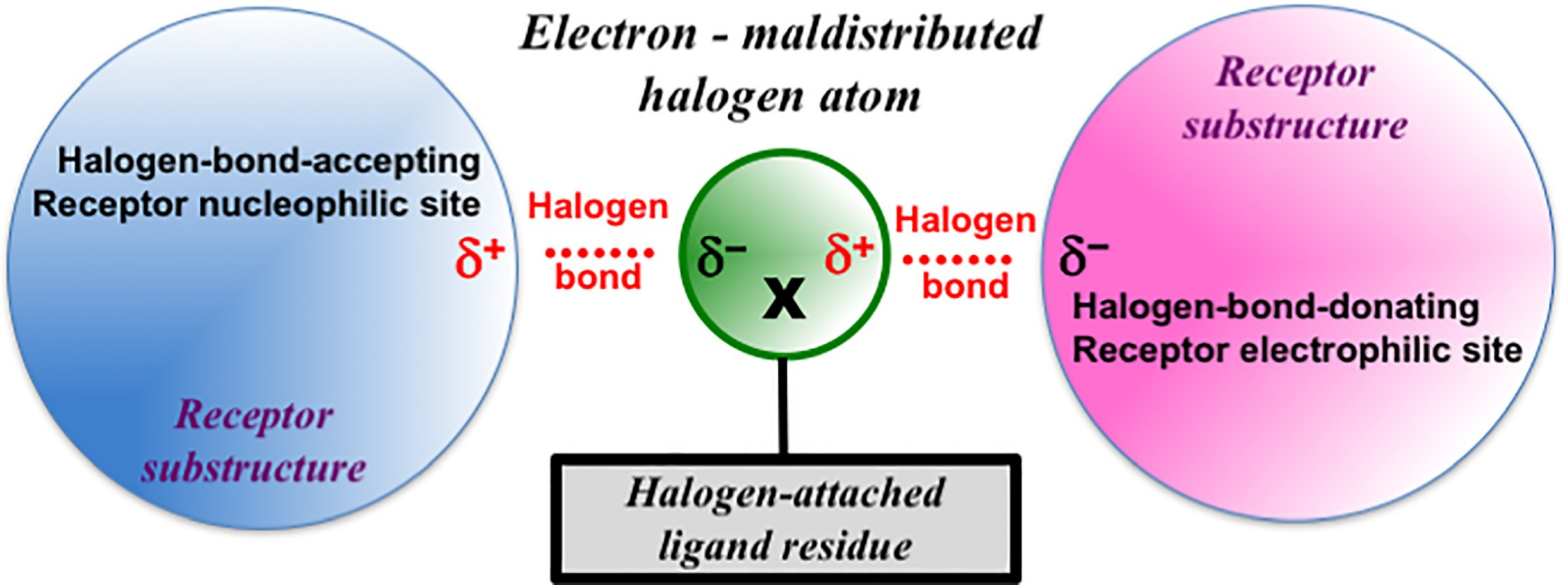
Schematic diagram of halogen bonding. Halogen bonds exist between the halogen atom X (F, Cl, Br, or I) and the electron-rich δ– and/or electron-poor δ+ sites in the receptor protein. The electrons of atom X in the ligand molecule are maldistributed to the electron-rich δ– and electron-poor δ+ portions.

The activity order of BPE-F < BPE-Cl < BPE-Br was well rationalized satisfactorily in the previous study [30] by the so-called halogen bond, the ascending strength order of which is F < Cl < Br < I [31]. As shown in Fig 7, when halogen atom X involved in the ligand molecule is under the London dispersion effect, X produces both electron-rich δ− and electron-poor δ+ portions on the same halogen atom surface [31]. The electron-poor δ+ portion is often referred to as the “sigma(σ)-hole” [44].

Anisotropic distribution of the electron density around covalently bound halogen atom would stimulate the interaction of halogen-containing ligand with the receptor counterpart(s). The resulting halogen bond between the ligand and the receptor molecules is described as like A····X — R, where A is the halogen-bond acceptor or donor and R is the halogen-attached ligand residue. The halogen bond (····) is non-covalent, while (—) is the carbon-halogen (C–X) covalent bond. The receptor ligand-binding site would be a halogen-bond-accepting nucleophilic site or a halogen-bond-donating electrophilic site (Fig 7). Thus, an ER, as a halogen-bond acceptor or donor, must possess such a nucleophilic end halogen-bond-accepting site or an electrophilic end halogen-bond-donating counterpart site. In order to interact with a halogen atom in any of the halogen-containing bisphenol compounds, ER must possess such counterpart(s) in its ligand-binding pocket.

The magnitude of electronegativity δ– or electropositivity δ+ depends on the dispersion force of halogen atoms and determines the strength of the interaction with the receptor δ+ or δ– site due to the resulting instantaneous dipole-induced dipole forces. It should be noted that larger and heavier atoms and molecules exhibit stronger dispersion forces than smaller and lighter ones [45], and this accounts very well for the activity order of BPE-F < BPE-Cl < BPE-Br [30]. Although the activity ascending order of BPE-F ≪ BPE-Cl ≲ BPAF < BPE-Br ≪ BPC should be explained fundamentally by the halogen bonding with the receptors as a fundamental driving force, the activity strength of BPAF and BPC must be explained by their some other structural characteristics.

### Structural effects of BPC on the enhanced receptor activity

The structural characteristics of BPAF and BPC are rather prominent because of the presence of characteristic structural elements, namely, because of the presence of the distal residues having geminal CF_3_ groups (BPAF) and Cl atoms (BPC). As to BPAF, two CF_3_ groups are present at the central bisphenol-connecting moiety (Fig 1). Since BPE-F has a single CF_3_ group, the order of BPE-F < BPAF was explained by such difference in the number of CF_3_ groups in their rigid conformation. Because all of these CF_3_ groups are tightly and rigidly fixed in the *sp*^3^ tetrahedral configuration, BPAF’s two CF_3_ groups would each hold a specific binding site, resulting in one additional binding site compared with BPE-F. We could justify consequently the order of BPE-F < BPE-Cl ≲ BPAF < BPE-Br [30].

**Fig 8:**
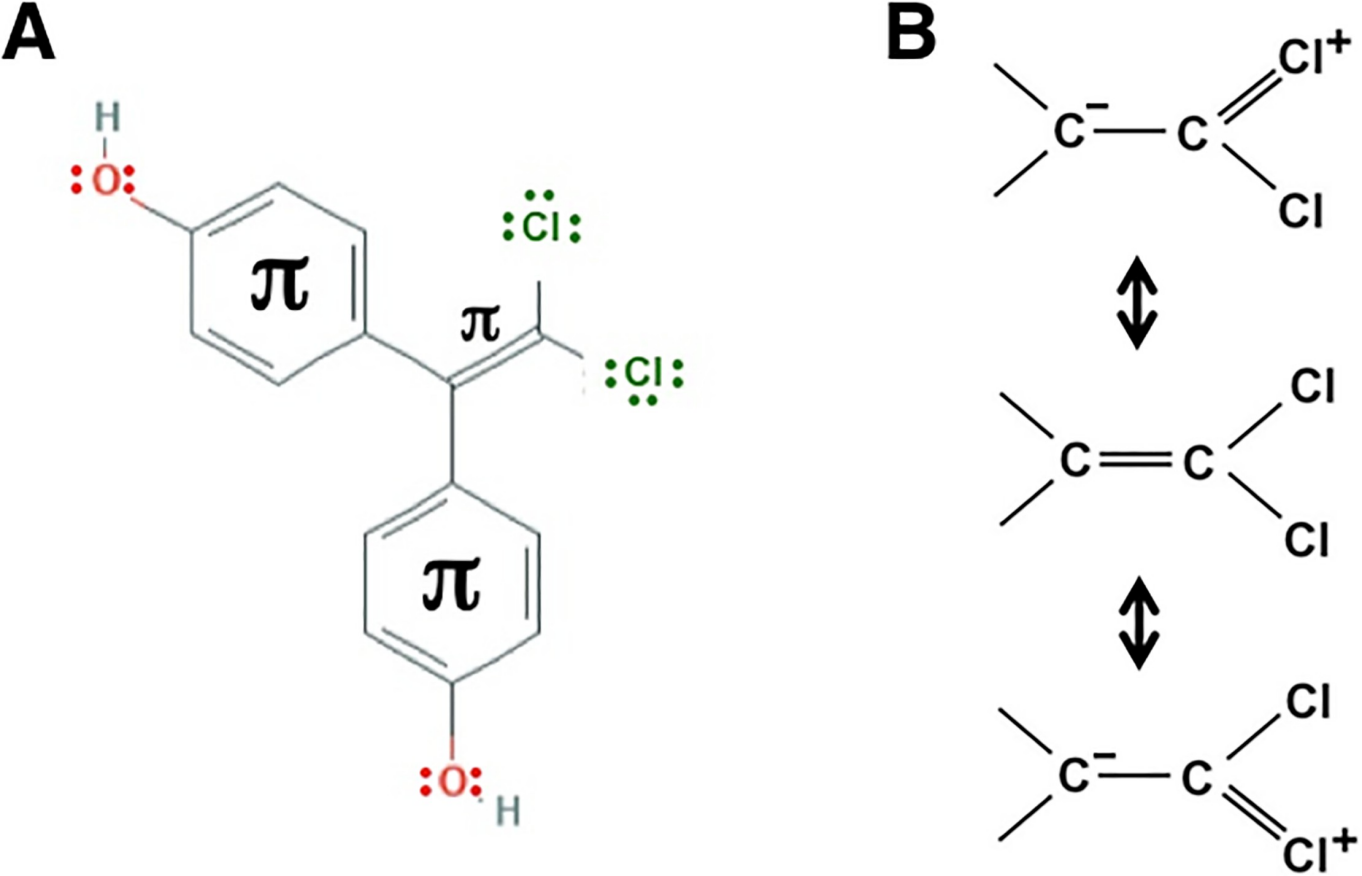
Interactive chemical structure models of BPC. Chemical constitution formulas are shown together with the electronic formula of chlorine and oxygen atoms (**A**), while the canonical forms or resonance structures are expressed for the vinylidene chloride moiety of BPC (**B**).

In the case BPC, two choline (Cl) atoms exist at the distal end in the central bisphenol-connecting moiety. These Cl atoms bind directly to the same *sp*^2^ C atom and are fixed in the C = C double bond coplanar as shown in Fig 8. Holding such a complete molecular rigidity or inflexibility, it is highly likely that each individual Cl atom in the central C = CCl_2_ moiety has a specific binding site, as mentioned above for the two CF_3_ groups of BPAF.

As to the central Cl-containing moiety, the BPC molecule is much smaller than that of BPAF (BPC = 96.77 Å^3^ is approximately 78% of BPAF = 124.44 Å^3^). Furthermore, because BPC possesses separate and independent Cl atoms instead of the very crowded tri-halogenated methyl CX_3_ groups present in BPE-F (CF_3_), BPE-Cl (CCl_3_), BPE-Br (CBr_3_), and BPAF (2 CF_3_) (Fig 1), Cl atoms of BPC would interact with receptor sites without any steric hindrance.

### The two n→π* transitions at the distal moieties of BPC molecule

All of the issues described above suggest that, for BPC’s highly strengthened interactions with ERs, BPC itself must have a molecular structure to make explainable the reason easily understandably. In addition to stereochemical characteristics, BPC appears to be in characteristic electronic structures, or electronic states. The molecular structure of BPC can be written as (HO–C_6_H_4_)_2_C = CCl_2_, in which two 4-hydroxyphenyl (= phenol) groups [(HO–C_6_H_4_)_2_], bind to the same carbon atom of *sp*^2^ C = C. At the same time, both of the Cl atoms bind to the other carbon atom of *sp*^2^ C = C, forming a 2,2-dichloroethylene (alias: vinylidene chloride) moiety (>C = CCl_2_) (Figs 1 and 8). As a result, the two sets of the phenol group and the Cl atom are fixed in a *trans* or *cis* location in the C = C double bond coplanar.

Each Cl atom possesses formally three lone pairs in the outermost shell (Fig 8A). Among these electron pairs, one exists in the 3*s* orbital and the other two exist in the 3*p* orbitals. Since the Cl atom is adjacent to the C = C double bond in BPC, the two 3*p* orbitals are filled with a total of four electrons and overlap structurally with the π orbitals of the C = C double bond. In addition, the four total electrons in the 3*p* orbitals of the Cl atom occupy the two molecular orbitals that have the lowest energies, namely, the two *nonbonding orbitals* (n). This places BPC in a resonance involving the overlap between the unfilled orbitals and a *filled* orbital, forming a resonance of >C = C–Cl ↔ >C^–^–C = Cl^+^ (Fig 8B).

**Fig 9:**
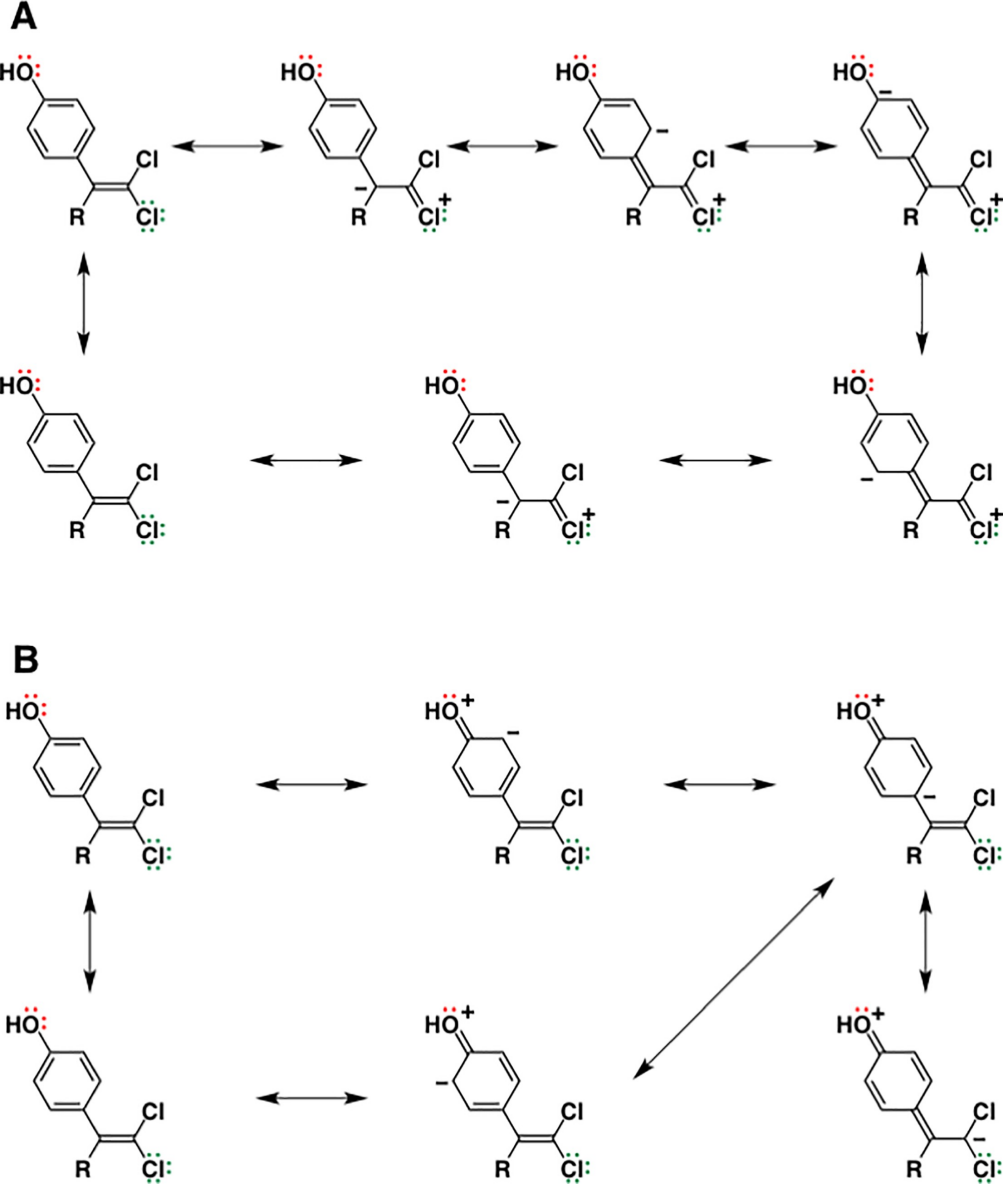
Resonance structures feasible for BPC. BPC is in a *trans* n_o_-π_benzene ring_-π_C = C_-n_Cl_ electron conjugation system. Resonance **A** shows the structures starting, at the top-left corner, from the n→π* transition of the chlorine atom 3*p* nonbonding orbital, whereas resonance **B** shows the structures starting from the n→π* transition of the oxygen atom 2*p* nonbonding orbital. **R** is another phenol group that is also involved in a similar *trans* n_o_-π_benzene ring_-π_C = C_-n_Cl_ electron conjugation. The resonance would take place also intersectingly in the *cis* arrangement.

In a resonance of the vinylidene chloride moiety (C = CCl_2_) (Fig 8B),

$$>C^{–}–C=Cl^{+}\left(–Cl\right)↔>C=CCl_{2}↔>C^{–}–C\left(–Cl\right)=Cl^{+}$$

the zwitterions >C^–^–C = Cl^+^(–Cl) and >C^–^–C(–Cl) = Cl^+^ themselves would be labile species interactive with the receptors. On the other hand, the resonance is a way of describing the molecular species, whether neutral molecules or ions, by a combination of several different contributing structures. The continuous transfers of electron pairs mutually relate these species to each other. In the case of BPC, among these three contributing structures of the vinylidene chloride moiety, the zwitterions would become a starting species for a new resonance in cooperation with the phenol group. In such a resonance, the electrons in the 3*p* nonbonding orbitals (n electrons) of Cl atoms would be involved in the n→π* transition [46], resulting in the participation of n electrons into the styrene-type π–π conjugation system (Fig 9A). Furthermore, it should be noted that the *cis*-*trans* cross-resonance would also be feasible, involving both the vinylidene chloride moiety (>C = CCl_2_) and the phenol groups.

The oxygen atom (O) on the phenol-hydroxy (O–H) group also has lone pairs in its outermost shell (Fig 8A). One of those two lone pairs exists in the 2*s* orbital, while the other exists in the 2*p* orbital. Since the O atom binds directly to the benzene π-conjugation system, the 2*p* n electrons are involved in the n→π* transition to conjugate with this π-system. Thus, 2*p* n electrons participate eventually in the styrene-type π–π conjugation system in the BPC molecule (Fig 9B).

### Intensified dispersion force in the prolonged n-π-π-n conjugation system

In a rigid coplanar conformation, the *trans*-located Cl atoms seem to be involved in the π-electron conjugation system between the C = C double bond and the aromatic benzene ring in the–C_6_H_4_–OH moiety. In this styrene-type π–π conjugation system, conjugated double bonds would consist of a total of eight flowable electrons ‘delocalized’. The styrene-type structure to which the two Cl atoms attach affords a big electrical characteristic to the BPC molecule, since the Cl atom consists of the lone pairs that make conjugation with the C = C double bond feasible. Collectively, the BPC of the structure (HO–C_6_H_4_)_2_C = CCl_2_ appears to have a prolonged conjugation system extending over the entire BPC molecule.

All of these results imply that the BPC molecule is in an extended or prolonged n-π-π-n conjugation system with a number of contributing structures (Fig 9). Apparently, this prolonged conjugation system extends over the entire BPC molecule. The important characteristics of this extended conjugation covering the n_o_, π_benzene ring_, π_C = C_, and n_Cl_ orbitals are that (i) the n_Cl_ and n_o_ electrons become delocalized and flowable in the whole BPC molecule and (ii) several contributing structures in the resonance possess plus-charged Cl or O atoms. Such species loading a plus-charged Cl atom would interact strongly with the electrophilic δ– sites or anionic sites (for example, a carboxylate ion–COO^–^) in the ligand-binding pocket of receptors ERα and ERβ. It is worth noting that, in the BPC molecule, there is another set of an extended n-π-π-n conjugation system involving the phenol group R and the Cl atom, as shown in Fig 9.

In relation to issue (i), it is feasible that the surface of the plain Cl atom with no plus-charge becomes locally electronegative δ– or electropositive δ+, or both (Fig 7). The dispersion force would bring about such polarization on the surface of the neat Cl atom because delocalized flowable electrons would easily gather together or get scattered even in the most distal Cl atom in an n-π-π-n conjugation system (Fig 9). Thus, the dispersion force *per se* can make easily polarize the originally electron-rich Cl atom, a total of 14 (= 2+6+2+4) outer-shell electrons being delocalized and flowable in an n-π-π-n conjugation system. The dispersion force would result in a much greater magnitude of electronegativity δ– or electropositivity δ+ on the Cl atom. Moreover, it should be noted that there are two sets of such a *trans* n-π-π-n electron conjugation arrangement in the BPC molecule (Figs 8A and 9), even with their possible *cis*-*trans* cross resonances. The total number of BPC resonance structures is thus more than 16. Although it is hard to figure out the structure of a resonance hybrid—that is, the combination of all resonance structures—the formation of δ–, δ+, or both sites on the Cl atom surface would elicit strong interactions between a BPC molecule and ERα and ERβ.

## Conclusion

The activity ascending order of BPE-F ≪ BPE-Cl ≲ BPAF < BPE-Br ≪ BPC is likely to be explained principally by the dispersion force of halogen atoms, but in cooperation with additional structural and electrical effects. In particular, the distal structure of BPC, namely, the vinylidene chloride >C = CCl_2_ moiety, is crucial to bring about the n→π* transition of the 3*p* nonbonding electrons of Cl atoms, letting those n electrons participate in the styrene-type π–π conjugation system. Subsequently, BPC’s central >C = CCl_2_ structure would cause the greatest electrophilic and structural influences to induce the strongest interactions with both ERα and ERβ. Since the bisphenols’ ligand-receptor interactions are based on the subsite interactions of the central halogen-containing moiety and the bilateral phenol groups with estrogen receptors, the central moiety is clearly the most important interacting structural element.

All of the halogen-containing bisphenol compounds tested in the present study were agonists for ERα but characteristic antagonists for ERβ, although those compounds appear to be accommodated completely inside the ligand-binding pocket of ERβ. The reason why these bisphenol compounds function as antagonists for ERβ must be that the binding to the binding pocket disturbs the interactions of ERβ with coactivator proteins, resulting in the formation of inhibitory conformation in DNA transcription. Obviously, *sp*^2^ >C = CCl_2_ of the BPC molecule is structurally essential in order to elicit such an inhibitory conformation, and thus BPC appears to be the most suitable model antagonist with supreme nucleophilic or electrophilic effects. We are now continuing our ERβ subsite explorations to learn which kinds of subsite interaction(s) do disturb the construction of activation conformation.
